# Associations of serum keratin 1 with thyroid function and immunity in Graves’ disease

**DOI:** 10.1371/journal.pone.0289345

**Published:** 2023-11-29

**Authors:** Chao-Wen Cheng, Wen-Fang Fang, Jiunn-Diann Lin

**Affiliations:** 1 Graduate Institute of Clinical Medicine, College of Medicine, Taipei Medical University, Taipei, Taiwan; 2 Traditional Herb Medicine Research Center, Taipei Medical University Hospital, Taipei Medical University, Taipei, Taiwan; 3 Cell Physiology and Molecular Image Research Center, Wan Fang Hospital, Taipei Medical University, Taipei, Taiwan; 4 Leader Clinic, Taipei, Taiwan; 5 Department of Internal Medicine, Division of Endocrinology, Shuang Ho Hospital, Taipei Medical University, New Taipei City, Taiwan; 6 Department of Internal Medicine, Division of Endocrinology and Metabolism, School of Medicine, College of Medicine, Taipei Medical University, Taipei, Taiwan; General Sir John Kotelawala Defence University Faculty of Medicine, SRI LANKA

## Abstract

**Background:**

Autoimmune thyroid disease (AITD) can cause enormous health burdens; however, trustworthy biomarkers in identifying the onset and progression of AITD are limited. In this study, we attempted to discover new potential serum biomarkers to discriminate AITD using mass spectrometry (MS).

**Methods:**

In the biomarker study cohort, 20 patients with Graves’ disease (GD), 20 patients with Hashimoto’s thyroiditis (HT), and 20 healthy controls were enrolled for a liquid chromatographic-tandem MS assessment. A novel biomarker, keratin 1 (KRT1), was selected for further evaluation in the validation cohort, including 125 patients with GD, 34 patients with HT, and 77 controls. Relationships of serum KRT1 with AITD-related immunomodulatory cytokines were also analyzed using enzyme-linked immunosorbent assays (ELISAs).

**Results:**

In the MS analysis, KRT1 was the single marker overexpressed in GD, while it was underexpressed in HT. In the ELISA analysis of the validation cohort, KRT1 was consistently upregulated in GD, while it was not downregulated in HT. There were significant associations of KRT1 levels with thyroid function in GD, AITD, and overall subjects. Additionally, a significant association of KRT1 levels with thyroid-stimulating hormone receptor antibody (TSHRAb) levels was observed. Moreover, there were significant associations of KRT1 with osteopontin (OPN) and B-cell activating factor (BAFF) levels in GD.

**Conclusions:**

Serum KRT1 levels were upregulated in GD and were associated with thyroid function and TSHRAb levels. Moreover, KRT1 was correlated with the BAFF and OPN levels in GD patients. Further molecular-based research to elucidate the role of KRT1 in the pathogenesis of AITD is needed.

## Introduction

Autoimmune thyroid disease (AITD), the most common organ-specific autoimmune disease (AID) worldwide, comprises two extremely diverse types: Graves’ disease (GD) and Hashimoto’s thyroiditis (HT) [1]. GD is driven by a unique pathogenic factor, the thyroid-stimulating hormone (TSH) receptor (TSHR) antibody (TSHRAb) which can recognize the TSHR in the thyroid, and ultimately stimulate thyroid hormone (TH) production [2,3]. On the contrary, different from GD driven by B-cell immunity, HT is chiefly triggered by cytotoxic T-cell pathogenesis, which subsequently contributes to diffuse immune cell infiltration over the thyroid and extensive thyrocyte death, and ultimately thyroid function decreases [4]. Although GD and HT are provoked by divergent mechanisms, individual genetic vulnerability and environmental exposure, including smoking, estrogen, infections, etc., are central contributors to the induction and progression of GD and HT [5].

For GD patients, chronic excessive TH exposure can increase the risk of arrhythmias, congestive heart failure, cardiovascular disease (CVD) mortality, osteoporosis, liver damage, etc. [6–8] In clinical settings, GD is initially treated with antithyroid drugs (ATDs); however, treatment outcomes are not encouraging with only approximately 50% remission and high recurrence rates (>50%) after discontinuing ATDs [9,10]. On the other hand, in addition to the decline in overall metabolism, HT patients with chronic hypothyroidism are at high risk of metabolic syndrome and CVDs, and most of them should be supplemented with levothyroxine (L-thyroxine) for the rest of their life [11,12]. As both GD and HT can cause health burdens, several approaches have been used to determine genetic variations and serum biomarkers in AITD patients, and early lifestyle interventions and treatment modalities targeting these molecules can be provided to slow disease progression and facilitate disease remission. Proteomics using mass spectrometry (MS) is an advanced technique to qualify and quantify differential expressions of serum proteins and discover diagnostic biomolecules between different groups with limited sample sizes. Nowadays, in clinical practice, it is widely used to detect new targets in various diseases, including tumors, CVDs, arthritis, etc. [13–15]. Meanwhile, only limited studies have screened for novel biomarkers in AITD patients by utilizing the MS technique. For instance, Jaber et al. and Lee et al. demonstrated serum metabolic profile alterations after ATD treatment in newly diagnosed hyperthyroid patients [16,17]. In addition, Dong et al. analyzed hyperthyroidism, hypothyroidism, and healthy subjects and discovered a novel lipid-metabolism marker regulated by TH that was involved in human metabolism [18]. Jiang et al. evaluated the differential proteomics of tears in patients with Grave’s orbitopathy and healthy subjects [19]. However, the above research mainly focused on associations of metabolic proteomic changes with thyroid function, and no study has explored immunomodulatory markers using the MS technique. In the present study, we initially identified a novel marker to differentiate GD, HT, and controls using an MS method in a biomarker study cohort, and subsequently validated the marker using an enzyme-linked immunosorbent assay (ELISA) method in a validation cohort. Through the discovery and verification process, keratin 1 (KRT1) was selected, and associations of KRT1 with thyroid function and the thyroid autoantibody in AITD were further evaluated. Moreover, associations of KRT1 with certain established immunomodulators that contribute to AITD, including B-cell activating factor (BAFF), osteopontin (OPN), interferon (IFN)-α, and IFN-β levels in GD were also assessed.

## Materials and methods

### Ethics statement

The study protocol was approved by the Joint Institutional Review Board of Taipei Medical University (TMU-JIRB-201414091), and all participants provided written informed consent prior to participation. This study was conducted in accordance with ethical standards of the *Declaration of Helsinki*.

### Subjects and study design

AITD blood samples were obtained by the Division of Endocrinology, Internal Department, Shuang Ho Hospital (New Taipei City, Taiwan) from January 2013 to September 2014, and included 125 GD and 34 HT patients. Blood samples of 77 participants without AID or AITD were obtained from the Health Screening Center of Shuang Ho Hospital from May to August 2014. Any participant was excluded if they were aged younger than 20 years, were pregnant, were alcoholic, or had a history of drug intoxication. GD was defined by one of the following criteria: (1) the presence of either reduced thyroid-stimulating hormone (TSH) level, either normal or increased free thyroxine (FT4), and the presence of circulating TSHRAb (121 patients); or (2) normal thyroid function and negative TSHRAb but with a prior diagnosis of GD by another hospital according to medical records (four patients). All 34 HT patients were confirmed to have the presence of either the anti-microsomal antibody (AmiA) or anti-thyroglobulin antibody (ATA) or both in conjunction with hypoechogenic thyroid parenchyma detected by a thyroid sonogram irrespective of normal or low thyroid function [20]. Among recruited subjects, 20 female GD patients, 20 HT patients, and 20 healthy controls were initially selected for the biomarker study. After determining protein concentrations in all samples, samples from the same group were pooled with the same amount of total protein levels for an MS analysis to unveil serum biomarker candidates to differentiate between AITD and the control group (Table 1). The top potential marker was chosen and further validated in all participants, including the 60 subjects in the initial MS study using an ELISA method.

**Table 1:** Demographic characteristics in the Graves’ disease (GD), Hashimoto’s thyroiditis (HT), autoimmune thyroid disease (AITD), and control groups in the biomarker study cohort.

Biomarker study cohort	Control	GD	HT	AITD
	(*n* = 20)	(*n* = 20)	(*n* = 20)	(*n* = 40)
Age (years)	41.6±11.2	42.1±10.5	47.7±13.3	44.9±12.2
Smokers (%)	5	0	5	2.6
Family history of thyroid disease (%)	0^b^^,^^c^^,^^d^	26.3^a^	25.0^a^	25.6^a^
FT4 (ng/dl)	-	3.33±2.48^c^	0.94± 0.14^b^	1.07 ± 0.56
log(TSH×10^4^) (μU/ml)	4.30±0.16^b^^,^^d^	2.13±0.46^a^^,^^c^	4.41±0.58^b^	3.27 ± 1.26^a^
TSHRAb (%)	-	62.5±24.9		-

Age is expressed as the mean±standard deviation.

^a^ *p* < 0.05 vs. the control group

^b^ *p* < 0.05 vs. GD

^c^ *p* < 0.05 vs. HT

^d^ *p* < 0.05 vs. AITD.

FT4, free thyroxine; TSH, thyroid-stimulating hormone; TSHRAb, thyroid-stimulating hormone receptor antibody.

GD patients were separated into two groups of inactive and active GD based on the TSH level at the time of sample collection. GD patients with high thyroid function, (TSH < 0.27 IU/L, the lower limit of the TSH reference range) were defined as having active GD, while those with TSH of ≥ 0.27 IU/L were defined as having inactive GD. Reporting of this study conformed to the STROBE statement along with references to the STROBE statement and broader EQUATOR guidelines [21].

#### Laboratory analyses

FT4 and TSH were determined by electrochemiluminescence immunoassay methods (Roche Diagnostica GmbH, Mannheim, Germany). Serum TSHRAb levels were quantified by a radioimmunoassay method (R.S.R., Cardiff, UK)). Data are expressed as the percentage blocking of I^125^-labeled TSH binding to the TSH receptor [22], and > 15% was considered positive.

In the first study to explore potential serum biomarker candidates, the serum protein preparation, including determining the protein concentration, digestion, and albumin and salt depletion were conducted as previously reported [14]. Subsequently, samples were analyzed using liquid chromatography tandem MS (LC-MS/MS) on a NanoAquity UPLC system (Waters, Milford, MA, USA) linked to an Orbitrap Elite Mass spectrometer (Thermo Electron, Waltham, MA, USA). The protocols were also conducted as described previously by Cheng et al. [14] The MS raw data were treated by PEAKS Studio (PEAKS 7, Bioinformatics Information, Ontario, Canada).

**Fig 1:**
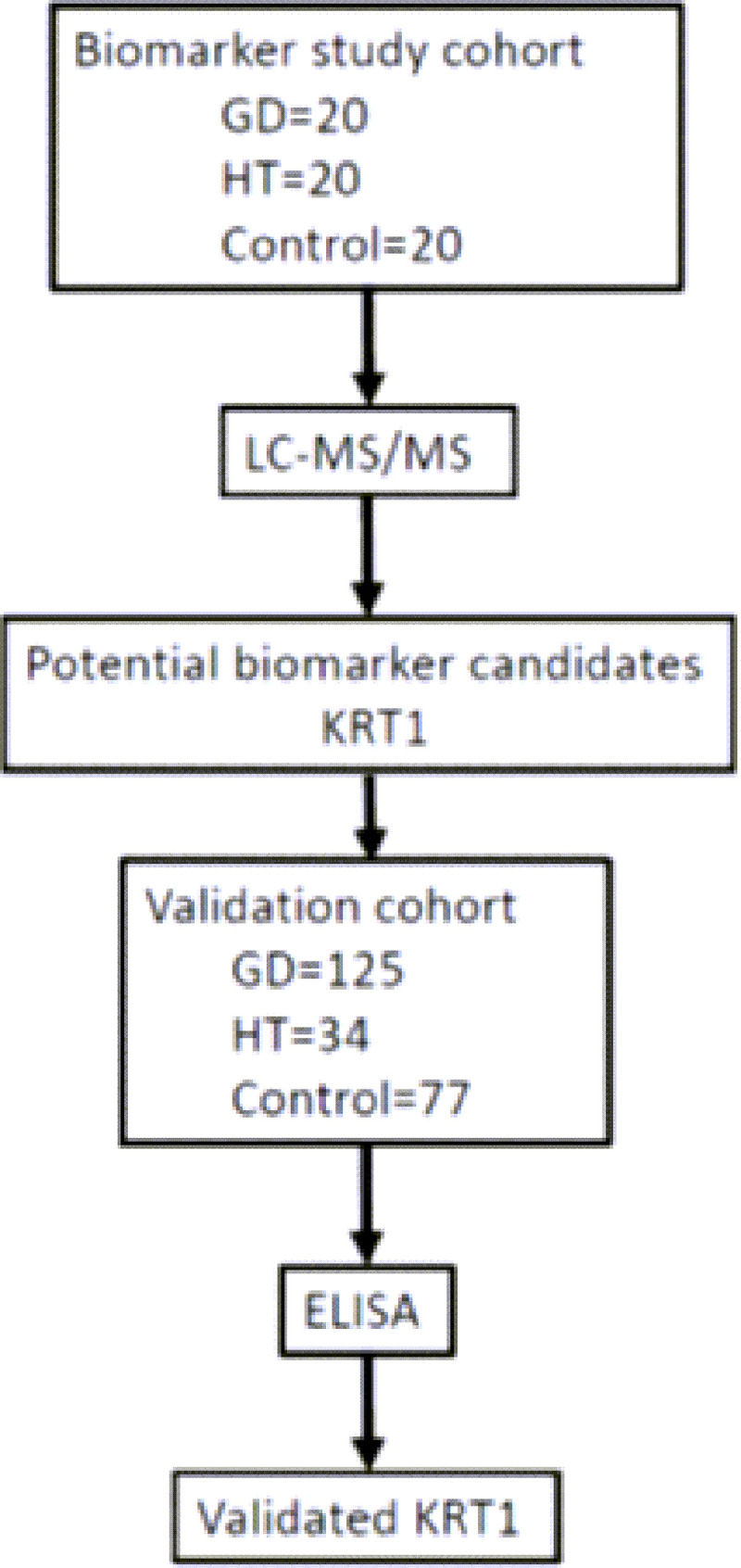
Experimental design of the study *p* < 0.05 indicates statistical significance. GD, Graves’ disease; HT, Hashimoto’s thyroiditis; LC-MS/MS, liquid chromatography tandem mass spectrometry; KRT1, keratin 1; ELISA, enzyme-linked immunosorbent assay.

Serum KRT1 levels were analyzed by an ELISA using a commercially available kit (Quantikine Human Keratin-1 Elabscience, Minneapolis, MN, USA). In addition, serum OPN, BAFF, IFN-α, and IFN-β levels were determined with ELISA kits (R&D Systems, Minneapolis, MN, USA) according to the manufacturer’s protocols. The experimental design of the study is illustrated in Fig 1.

### Statistical analysis

All statistical analyses were performed using IBM SPSS Statistics 19.0 (Armonk, NY, USA). Quantitative values are presented as the mean ± standard deviation (SD). All data were tested for a normal distribution using the Kolmogorov-Smirnov test and homogeneity of variance using Levene’s test. Among the data, FT4, TSH, IFN-α, and IFN-β were right-skew distributed and thus were logarithmically transformed. Some hyperthyroid patients had undetectable TSH levels (for instance: < 0.008 IU/ml); therefore, for those patients, the lowest detection limit value (0.008 IU/ml) was regarded as the TSH value. An independent *t*-test was used to compare differences in clinical variables between two groups. A χ^2^ test was used to assess differences in the GD, HT, and AITD groups with the control group. A one-way analysis of variance (ANOVA) was used to compare differences in clinical parameters among the GD, HT, and control groups. The Bonferroni test was used for post-hoc examinations. A χ^2^ test or Fisher’s exact test was also used to assess differences in categorical data between two groups. In order to eliminate the bias of demographic variables on the KRT1 level, a multiple regression analysis was used, and standardized coefficients (β) were obtained. We took FH of thyroid disease and smoking, together with either GD or active GD or AITD, as independent variables with KRT1 as the dependent variable. All statistical tests were two-sided, and a *p* value of < 0.05 was considered significant.

## Results

### Demographic data of the GD, HT, AITD, and control groups

In the biomarker study cohort, GD, HT, and AITD patients had greater prevalences of a family history of thyroid disease (FH) than the control group (Table 1). Demographic characteristics of the validation cohort are shown in Table 2. HT patients were older than GD patients and controls, and the proportion of women in the HT group was higher than those in the GD and control groups. GD, HT, and AITD patients had a greater prevalence of a family history of thyroid disease (FH) than did healthy controls. Patients in the GD group had a higher prevalence of a smoking habit than did those in the HT and control groups. On the other hand, the percentage with a smoking habit was higher in the AITD group (26.1%) than in the control group (13.0%).

**Table 2:** Demographic characteristics in Graves’ disease (GD), Hashimoto’s thyroiditis (HT), autoimmune thyroid disease (AITD), and control groups in the validation cohort.

Validation cohort	Control	GD	HT	AITD
	(*n* = 77)	(*n* = 125)	(*n* = 34)	(*n* = 159)
Age (years)	41.2±10.1^c^	41.6±11.7^c^	49.1±14.2^a^^,^^b^	43.2±12.6
Gender (women, %)	59.7^c^	59.2^c^	82.4^a^^,^^b^	64.2
Smokers (%)	13.0^b^^,^^d^	30.1^a^^,^^c^	11.8^b^	26.1^a^
Family history of thyroid disease (%)	2.6^b^^,^^c^^,^^d^	30.2^a^	17.6^a^	27.3^a^
FT4 (ng/dl)	-	1.59±1.54	1.03± 0.39	1.07 ± 0.56
log(TSH×10^4^) (μU/ml)	4.22±0.24^b^^,^^d^	3.34±1.07^a^^,^^c^	4.31±0.60^b^	3.54 ± 1.07^a^
TSHRAb (%)	-	38.9±23.3		-

Age is expressed as the mean±standard deviation.

^a^ *p* < 0.05 vs. the control group

^b^ *p* < 0.05 vs. GD

^c^ *p* < 0.05 vs. HT

^d^ *p* < 0.05 vs. AITD.

FT4, free thyroxine; TSH, thyroid-stimulating hormone; TSHRAb, thyroid-stimulating hormone receptor antibody.

### Differential serum KRT1 levels in the GD, HT, and control groups

**Fig 2:**
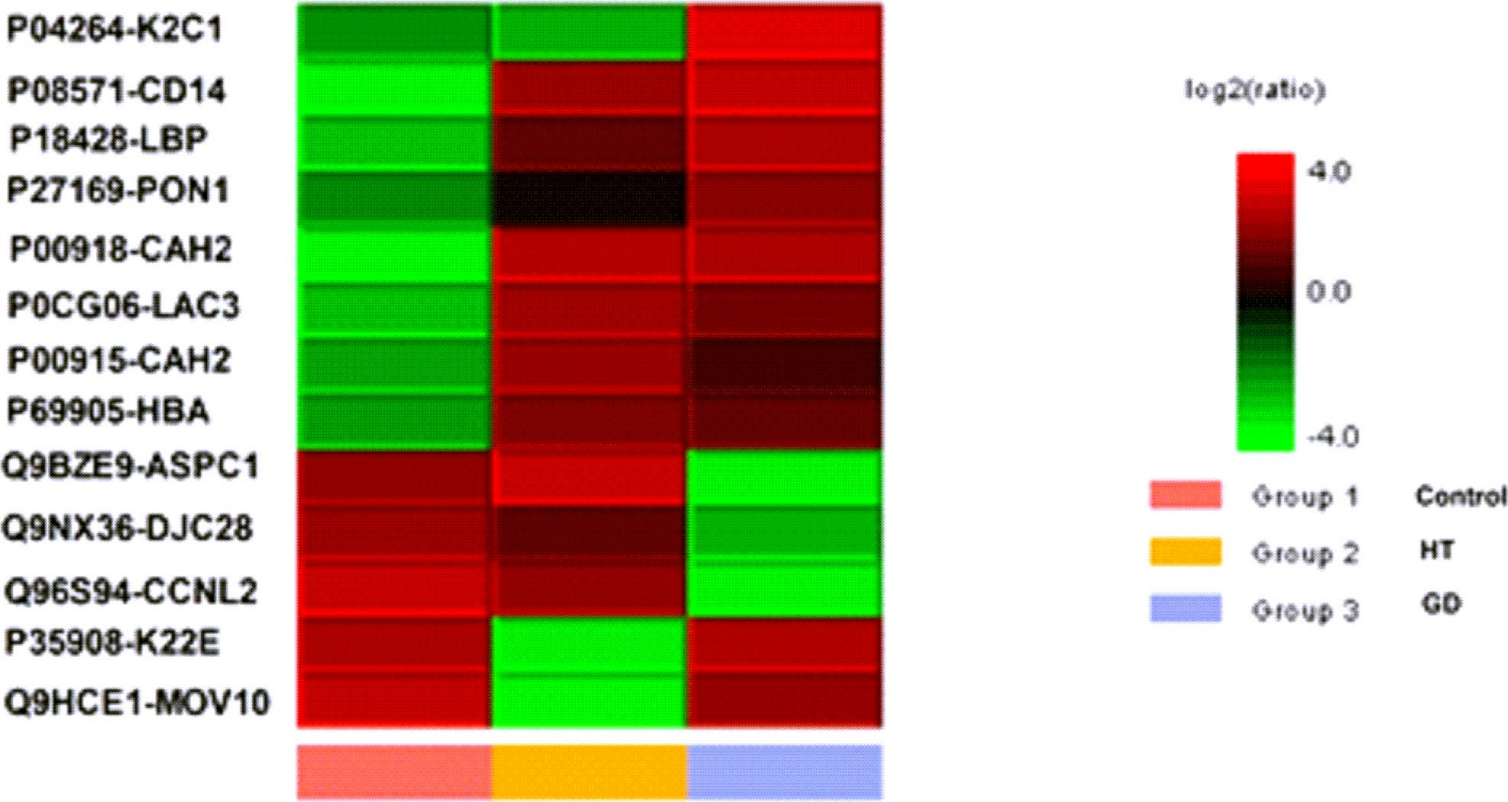
Differential protein levels among the control, Hashimoto’s thyroiditis (HT), and Grave’s disease (GD) groups using mass spectrometry.

To discover a potential target to discriminate among GD (hyperthyroidism), HT (hypothyroidism), and normal thyroid function, we initially performed a serum proteomic analysis using LC-MS/MS in the biomarker cohort. Fig 2 shows a heatmap created by PEAK7 software among the GD, HT, and control groups, and the Individual protein name and function are shown in Table 3.

**Table 3:** Individual protein name and function in Fig 2 (as analyzed by mass spectrometry).

Number	Protein name	Function
P04264-K2C1-human	Keratin, type II cytoskeleton 1 (KRT1)	Regulates skin or intestinal and skin integrity and forms epithelial barrier.
P08571-CD14-human	Monocyte differentiation antigen CD14	Mediates immune responses to bacterial lipopolysaccharide.
P18428-LBP-human	Lipopolysaccharide-binding protein	Plays a role in innate immune responses, promotes the release of cytokines.
P27169-PON1-human	Serum paraoxonase	Transfers proteins between phospholipid surfaces.
P00918-CAH2-human	Carbonic anhydrase 2	Essential for bone resorption and osteoclast differentiation.
P0CG06-LAC3-human	Immunoglobulin lambda-3 chain C regions	Constant region of immunoglobulin light chains.
P00915-CAH1-human	Carbonic anhydrase 1	Reversible hydration of carbon dioxide.
P69905-HBA-human	Hemoglobin subunit alpha	Is involved in oxygen transport from the lungs to various peripheral tissues.
Q9BZE9-ASPC1-human	Tether containing UBX domain for glucose transporter 4 (GLUT4)	Tethering protein that sequesters GLUT4-containing vesicles in the cytoplasm in the absence of insulin. Modulates the amount of GLUT4 that is available at the cell surface.
Q9NX36-DJC28-human	DnaJ homolog subfamily C member 28	May have a role in protein folding or as a chaperone.
Q96S94-CCNL2-human	Cyclin-L2	Is involved in pre-messenger (m)RNA splicing. May induce cell death, possibly by acting on the transcription and RNA processing of apoptosis-related factors.
P35908-K22E-human	Keratin, type II cytoskeletal 2 epidermal	Associated with keratinocyte activation, proliferation. and keratinization. Plays a role in the establishment of the epidermal barrier on plantar skin.
Q9HCE1-MOV10-human	Helicase MOV-10	5’ to 3’ RNA helicase contributing to UPF1 mRNA target degradation by translocation along 3’ untranslated regions.

KRT1, keratin1 (keratin, type II cytoskeleton 1).

**Fig 3:**
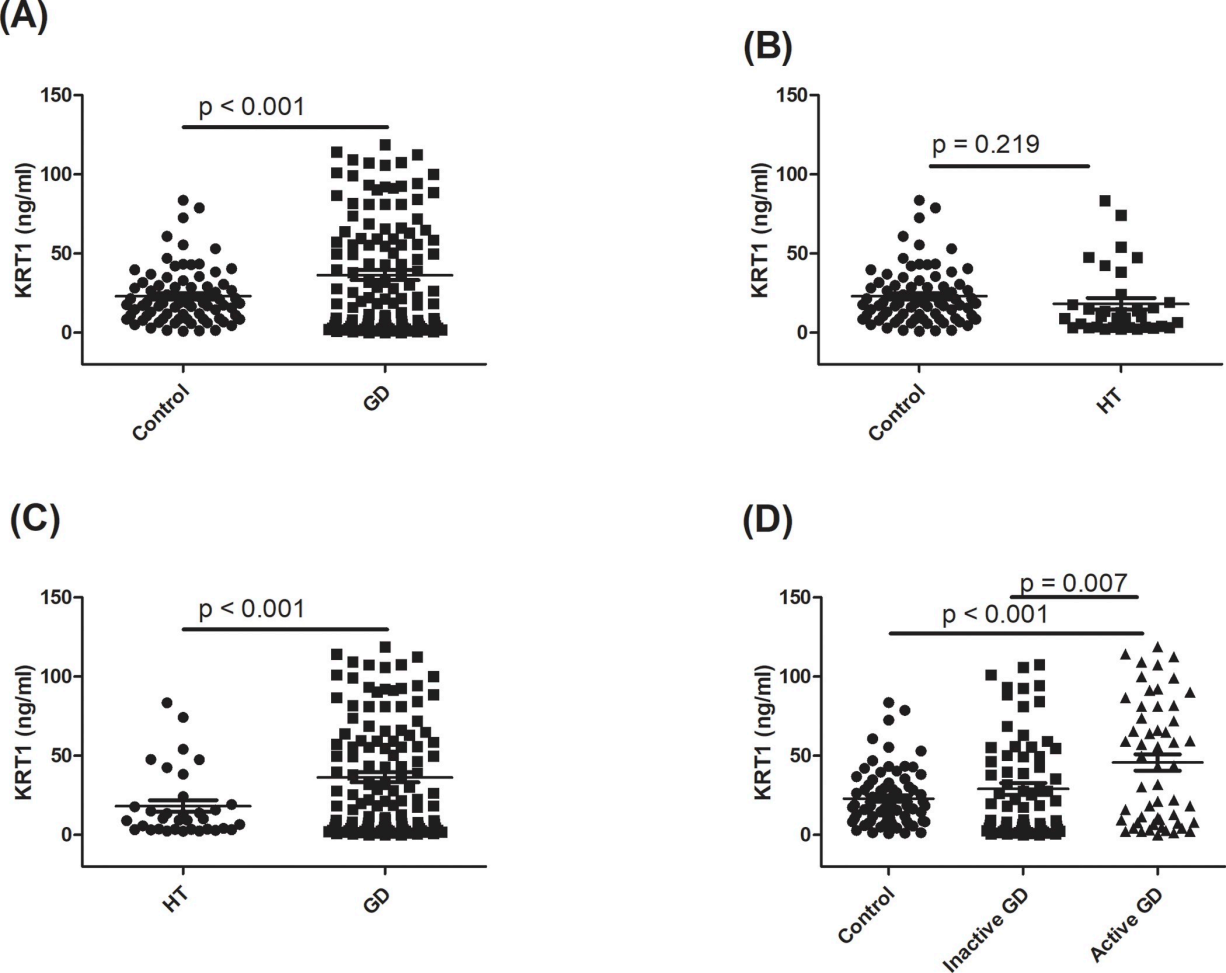
Serum keratin 1 (KRT1) protein measured by an enzyme-linked immunosorbent assay method among Graves’ disease (GD), Hashimoto’s thyroiditis (HT), and control subjects. *p* < 0.05 indicates statistical significance.

The serum KRT1 level was the only biochemical molecule that exhibited downregulation in HT and upregulation in GD patients in these limited samples. It was previously documented that KRT1 is tightly regulated by TH in keratinocytes, and can participate in skin manifestations in hyperthyroidism and hypothyroidism [23,24]. Accordingly, KRT1 was selected as a pilot serum biomarker to discriminate between AITD and normal subjects. To validate the proteomic results, serum KRT1 was further determined using an ELISA method in the validation cohort. Data showed that serum KRT1 was higher in the GD group than in the control group, while the KRT1 level was slightly reduced in the HT group compared to the control group, but this did not reach statistical significance (Fig 3A and 3B, respectively). On the other hand, the serum KRT1 level in the GD group was also higher than that in the HT group (*p* < 0.001, Fig 3C). In addition, there were significant differences in serum KRT1 levels between patients with inactive GD and active GD (*p* = 0.007), and between active GD patients and the controls (*p* < 0.001, Fig 3D). A multiple linear regression analysis was used to evaluate the influence of demographic variables on KRT1 levels, and results are shown in Table in S1 File. We confirmed that either GD, active GD, or AITD was still retained in the regression model in predicting KRT1 levels after adjusting for these variables.

### Associations of serum KRT1 levels with FT4, TSH, and TSHRAb

**Fig 4:**
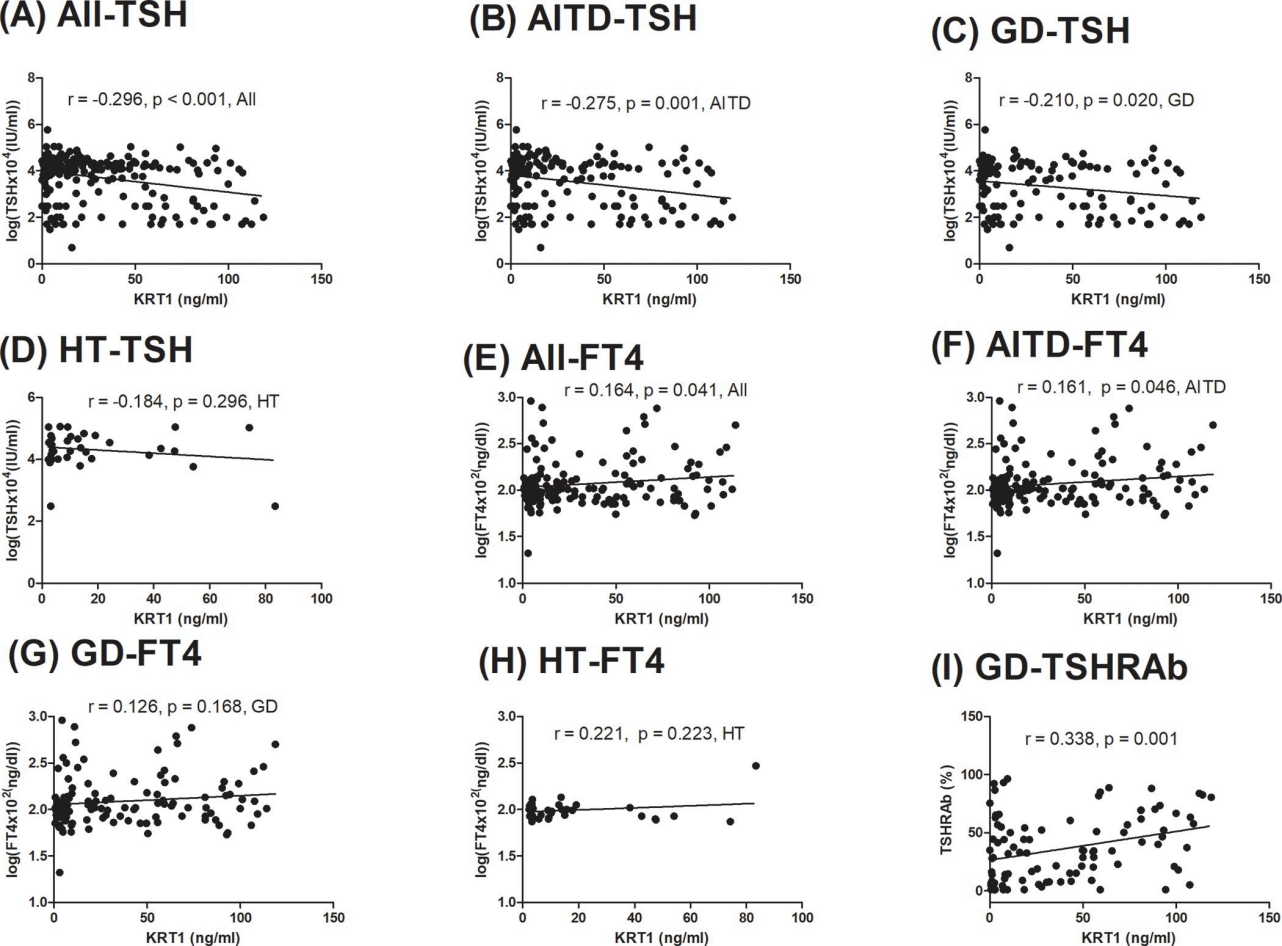
Associations of serum keratin1 (KRT1) with thyroid-stimulating hormone (TSH), and free thyroxine (FT4) in all, autoimmune thyroid disease (AITD), Graves’ disease (GD), and Hashimoto’s thyroiditis (HT) subjects, and the association of KRT1 with the TSH receptor antibody (TSHRAb) in GD.

There were associations of serum KRT1 with TSH levels in all, AITD, and GD subjects (*r* = -0.296, *p* < 0.001; *r* = -0.275, *p* = 0.001; *r* = -0.210, *p* = 0.020, Fig 4A–4C, respectively), but not in HT subjects (*r* = -0.184, *p* = 0.296, Fig 4D). In addition, there was an association of serum KRT1 levels with FT4 in all subjects and the AITD group (*r* = 0.164, *p* = 0.041; *r* = 0.161, *p* = 0.046, Fig 4E and 4F, respectively), while there was no association of serum KRT1 levels with FT4 in the GD or HT groups (*r* = 0.126, *p* = 0.168; *r* = 0.221, *p* = 0.223, Fig 4G and 4H, respectively). Moreover, no significant correlation existed between serum KRT1 and TSH levels in the normal controls (*r* = -0.201, *p* = 0.135, Figure in S1 File). In addition, there was also a significant association of serum KRT1 levels with TSHRAb levels in GD patients (*r* = 0.338, *p* = 0.001, Fig 4I).

### Associations of serum KRT1 levels with OPN and BAFF in GD patients

**Fig 5:**
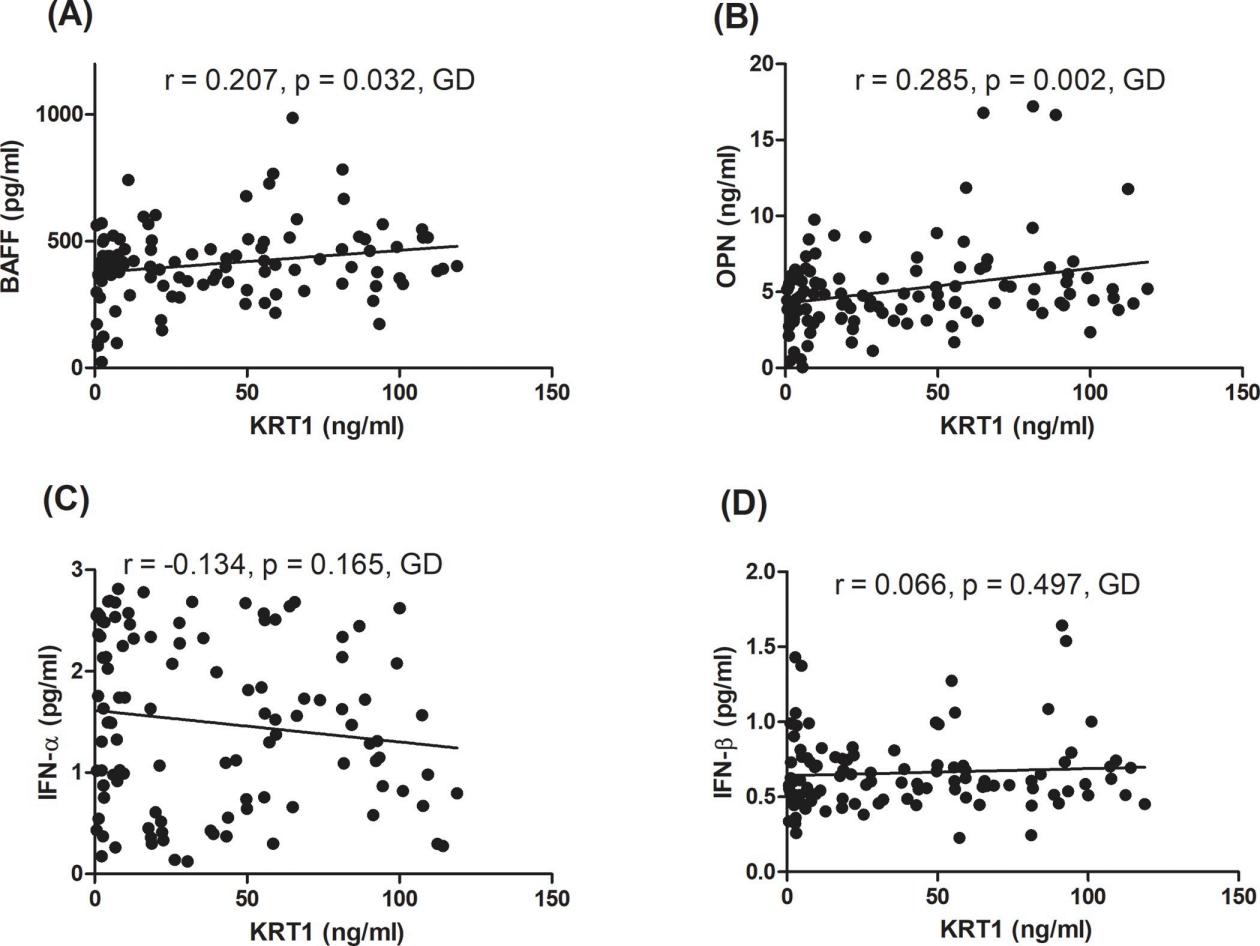
Associations of serum keratin 1 (KRT1) with B-cell activating factor (BAFF), osteopontin (OPN), interferon (IFN)-α, and IFN-β levels in Graves’ disease. *p* < 0.05 indicates statistical significance.

As there was a significant association of serum KRT1 with TSHRAb levels in GD patients, which implied that serum KRT1 could contribute to the pathogenesis of GD, we further evaluated correlations of serum KRT1 with certain immunomodulators, including OPN, BAFF, IFN-α, and IFN-β. There were significant associations of serum KRT1 with BAFF levels in GD subjects (*r* = 0.207, *p* = 0.032, Fig 5A). In addition, we observed a significant correlation between serum KRT1 and OPN levels in GD patients (*r* = 0.285, *p* = 0.002; Fig 5B). Meanwhile, there were no significant correlations between serum KRT1 and IFN-α, or between serum KRT1 and IFN-β levels in either the GD or control groups, and results are shown in Fig 5C and 5D.

## Discussion

In this study, we initially discovered that KRT1 was a potential serum biomarker to distinguish among GD, HT, and control subjects using a promising approach of the LS-MS/MS technique. Using an ELISA method, we confirmed that serum KRT1 levels were higher in patients with GD than those in the HT and control groups, while KRT1 was not significantly reduced in HT. In addition, we observed that serum KRT1 levels were associated with TSHRAb levels in GD patients. These interesting findings implied that in addition to the association of serum KRT1 with thyroid function, KRT1 also potentially exerts a role in the pathogenesis of GD. Moreover, we demonstrated that serum KRT1 levels were associated with serum OPN and BAFF levels but not with IFN-α or IFN-β levels in GD. These findings suggested that in GD patients, possible coordinated effects of KRT1 with BAFF and OPN could participates in the pathogenesis of GD. These results could offer important information in understanding the role of KRT1 in the pathogenesis of AITD.

Patients with thyroid dysfunction usually have certain skin manifestations, for instance, hyperthyroid patients may have skin thinning, while those with hypothyroidism have coarse skin [25]. TH can promote keratinocyte proliferation and dermal fibroblast hyperplasia, and directly regulate multiple *KRT* gene expressions by acting on upstream TH-response elements [26–30]. KRT1, one of the KRTs, is a member of the intracellular component of intermediate filaments of the cytoskeleton, and KRT1 expression is highest in the skin and appendages among all tissues [31,32]. Therefore, it is possible that in hyperthyroidism, elevated TH promotes excessive KRT1 production in the skin, is subsequently released into the blood circulation, and ultimately leads to a rise in serum KRT1 levels. However, in addition to skin tissues, KRT1 can also be detected in multiple cell types, including blood cells, the thyroid gland, etc., and serum KRT1 alterations might also be attributed to increased production by TH by the thyroid gland or blood cells. In contrast, contradictory to the findings of the MS analysis, our validated cohort showed no association of serum KRT1 with HT. The discrepant results in the HT group between the ELISA and LS-MS analysis could be attributed to different sample preparation, principles of the quantification methods, and sample sizes. In the current study, blood samples in the MS study were pooled for further proteomics analysis, while KRT1 was measured in individual samples using an ELISA, and differences in individual KRT1 expressions present in the ELISA could have been obscured by pooling samples in the MS analysis. In addition, the principle of recognizing proteins using the MS method is to break down the protein into several amino acids and polypeptide contents of a distinct protein, which entirely differs from the ELISA method of identifying the presence of intact proteins. The divergence between the two techniques may also have contributed to the discrepancy. Moreover, only 34 HT patients were included in the study; recruiting more HT patients could have further validated the data and made our results more convincing and valuable [33–35].

Interestingly, in addition to possibly being regulated by TH, serum KRT1 levels were demonstrated to be linked to TSHRAb levels, which implied that KRT1 could alternatively be engaged in the pathogenesis of GD. The skin is the first-line barrier to protect the body from external injury, such as ultraviolet light, infections, trauma, etc. [36] In addition to resident immunocytes which maintain skin immunological homeostasis, keratinocytes themselves can act as immune sensors, and can modulate skin immune and inflammatory reactions through exciting cytokine and chemokine release, promoting immunological and major histocompatibility complex (MHC) molecule expressions, recruiting immune cells, and further promoting inflammatory and immune processes [36–42]. However, it is still unclear whether the major intracellular cytoskeletal molecule, KRT1, participates in immune and inflammatory processes in the skin and other tissues. Roth et al. showed that KRT1 may be involved in supporting the intact skin barrier and regulating inflammatory conditions by way of IL-18 [43]. Moreover, Guo et al. disclosed that KRT1 in vessel walls could be a potential target antigen of autoimmune processes in allograft rejection disease [44]. Limited studies have highlighted the potential role of KRT1 in initiating and regulating autoimmunity. As mentioned above, although KRT1 is most abundant in skin tissues, KRT1 may be also present in the blood and thyroid gland [31,32]. Therefore, it also probable that either dysregulated thyroid or blood KRT1 can exhibit breakthrough immune tolerance and subsequently trigger thyroid autoimmunity. Well-designed molecular-based research to dissect the actual mechanism of KRT1 in thyroid autoimmunity is needed.

Another interesting finding of the present study was that we showed that serum KRT1 was associated with other key immune players, BAFF and OPN. BAFF can especially prolong B-cell survival, is crucial for B-cell growth, evolution, and differentiation, and ultimately facilitates antibody formation. On the other hand, OPN, an essential bone-remodeling-associated protein, mainly promotes T-cell, macrophage, dendritic cell, and B-cell activation, and subsequently modifies T-cell-mediated and B-cell-mediated immunity. Either imbalanced BAFF or OPN can escape immune tolerance and result in AID development [45–50]. Moreover, in our recent publications, we demonstrated that serum BAFF and OPN levels were upregulated in GD, were associated with TSHRAb and thyroid function, and suggested that both BAFF and OPN could exhibit remarkable roles in inducing GD and modulating disease progression [51,52]. Correlations of KRT1 with BAFF and OPN levels indicate that the existence of KRT1 with either BAFF or OPN might be coordinated. Evidence showed that BAFF is co-expressed with certain KRTs and respectively participates in allograft reactions and inflammatory skin diseases in the kidneys and skin tissues [53,54]. On the other hand, other research demonstrated that OPN was detected in keratinocytes, and might participate in keratinocyte differentiation and modulate T-cell excitation [55,56]. All of these earlier reports strongly linked KRT1 to both BAFF and OPN expressions. As mentioned above, keratinocytes can work as both immune sentinels and effectors, to regulate inflammatory and immune responses; thus, it would not be surprising that there is interplay between KRT1 and either BAFF or OPN, which are two powerful immunoregulatory markers. Based on these observations, we postulated that KRT1, OPN, and BAFF are maintained at low concentrations in normal subjects. After GD develops, KRT1-BAFF and KRT1-OPN immunoregulatory signals are both provoked, and accordingly stimulate TSHRAb synthesis. However, the precise causal relationships and mechanisms between KRT1 and BAFF, and between KRT1 and OPN are unknown. Further molecular-based studies are needed to elucidate this issue. On the other hand, we also analyzed associations of KRT1 with IFN-α and IFN-β, two other important immunoregulators in GD; nevertheless, we observed no associations of KRT1 with IFN-α or IFN-β. These observations indicate that the impact of interaction between KRT1 and type 1 IFN in inducing or regulating disease progression of GD is limited.

In the present study, we demonstrated that serum KRT1 was higher in GD, and KRT1 was associated with thyroid function and TSHRAb levels. In addition, we are the first to demonstrate significant associations of serum KRT1 levels with BAFF and OPN proteins in GD patients. However, certain limitations in the present study should be pointed out. First, the present study was only a cross-sectional study, and the precise mechanisms of these associations of KRT1 with thyroid function, TSHRAb, BAFF, and OPN are unclear. Future well-designed studies using both in vitro and in vivo experiments to dissect the pathophysiological mechanism of KRT1 in inducing or regulating AITD are required. Second, as aforementioned, the finding that KRT1 is downregulated in HT as identified by the LS-MS/MS method could not be successfully validated using the ELISA method. The discrepant results could be partially attributed to the limited sample size of HT patients. Further study to recruit more HT patients could help clarify the discordant results. Finally, possible causes of elevated serum KRT1 in hyperthyroidism could mainly be derived from a skin origin; however, thyroid and peripheral blood white blood cells (PBMCs) could be other potential sources. Those tissues were not collected for the KRT1 mRNA analysis in the current study, and the main source of elevated serum KRT1 in GD remains unclear. It is our future plan to gather mouse serum, skin, thyroid, and peripheral white blood cells at the same time, to quantify KRT1 transcripts of these tissues using the mouse thyroiditis model built by our laboratory, to elucidate the possible origin of elevated serum KRT1 stimulated by the TH [57].

In conclusion, serum KRT1 levels were upregulated in GD and were associated with thyroid function and TSHRAb levels. In addition, serum KRT1 levels were correlated with BAFF and OPN levels in GD patients. Further molecular-based research to elucidate the role of KRT1 in the pathogenesis of AITD is needed.

## Supporting information

S1 File Supplementary figure and table. (DOCX)

S2 File Dataset. (XLS)

## Abbreviations

AID: autoimmune disease
AITD: autoimmune thyroid disease
AmiA: antimicrosomal antibody
ATA: antithyroglobulin antibody
BAFF: B-cell activating factor
CVD: cardiovascular disease
ELISA: enzyme-linked immunosorbent assay
E2: estrogen
GD: Graves’ disease
HT: Hashimoto’s thyroiditis
IFN: interferon
KRT1: keratin 1
LC-MS/MS: liquid chromatography tandem mass spectrometry
MS: mass spectrometry
OPN: osteopontin
TH: thyroid hormone
TSH: thyroid-stimulating hormone
TSHR: thyroid-stimulating hormone receptor
TSHRAb: thyroid-stimulating hormone receptor antibody
