# Correction: Associations of serum keratin 1 with thyroid function and immunity in Graves’ disease

**DOI:** 10.1371/journal.pone.0335428

**Published:** 2025-10-24

**Authors:** Chao-Wen Cheng, Wen-Fang Fang, Jiunn-Diann Lin

In the Ethics statement subsection of the Materials and Methods, there is an error in the first sentence. The correct sentence is: The study protocol was approved by the Joint Institutional Review Board of Taipei Medical University (TMU-JIRB-201404091), and all participants provided written informed consent prior to participation.
