# Supplementary material for: Associations of serum keratin 1 with thyroid function and immunity in Graves’ disease
Source: PLoS One. 2023 Nov 29;18(11):e0289345. doi: 10.1371/journal.pone.0289345 (PMC10686460; doi:10.1371/journal.pone.0289345)

Supplementary Table 1: Multivariable linear regression analysis of either Graves’ disease (GD), active Graves’ disease (active GD), or autoimmune thyroid disease (AITD) and the significant demographic factors with KRT1 levels

| KRT1 level (ng/ml) | Beta | P value |
| --- | --- | --- |
| GD | 0.243 | 0.001 |
| FH of thyroid disease (FH) | -0.115 | 0.122 |
| Smoking habit (%) | 0.181 | 0.011 |
| KRT1 level (ng/ml) |  |  |
| Active GD | 0.450 | <0.001 |
| FH of thyroid disease (FH) | -0.369 | 0.806 |
| Smoking habit (%) | -0.183 | 0.042 |
| KRT1 level (ng/ml) |  |  |
| AITD | 0.159 | 0.022 |
| FH of thyroid disease (FH) | -0.067 | 0.330 |
| Smoking habit (%) | 0.181 | 0.007 |

Beta, standardized coefficients;

Supplementary Figure 1:


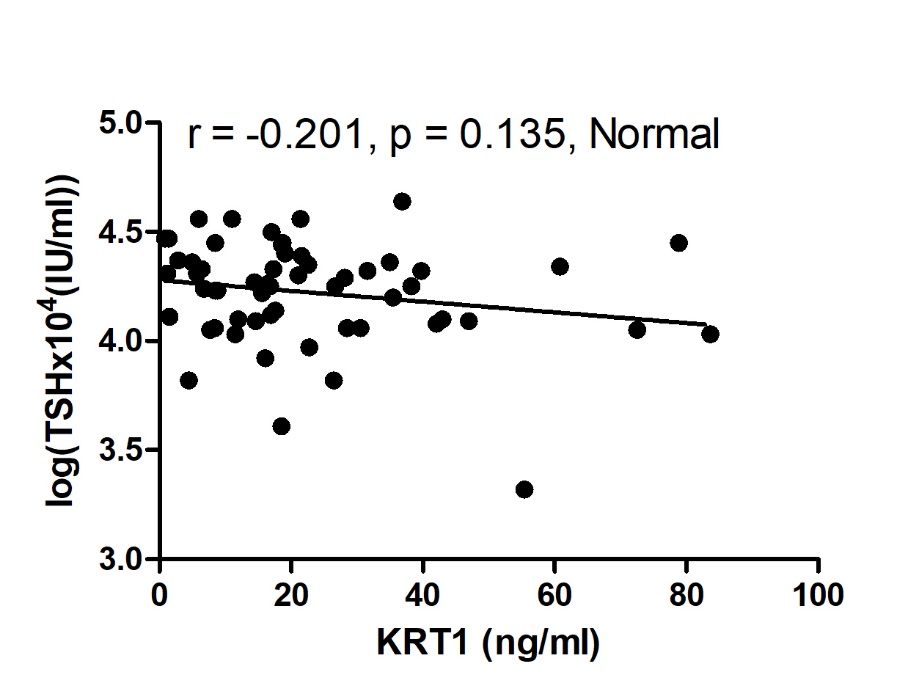

Supplement: S1 File — (DOCX) [file pone.0289345.s001.docx]
